# Impact of the Double Mutants on Spike Protein of SARS-CoV-2 B.1.617 Lineage on the Human ACE2 Receptor Binding: A Structural Insight

**DOI:** 10.3390/v13112295

**Published:** 2021-11-17

**Authors:** Mohd Imran Khan, Mohammad Hassan Baig, Tanmoy Mondal, Mohammed Alorabi, Tanuj Sharma, Jae-June Dong, Jae Yong Cho

**Affiliations:** 1 Department of Internal Medicine, Gangnam Severance Hospital, Yonsei University College of Medicine, Gangnam-gu, Seoul 120-752, Korea; imranimran.amu@gmail.com; 2 Department of Family Medicine, Gangnam Severance Hospital, Yonsei University College of Medicine, Gangnam-gu, Seoul 120-752, Korea; mhbaig@yonsei.ac.kr (M.H.B.); tanush84@gmail.com (T.S.); 3 Laboratory Medicine, Institute of Biomedicine, University of Gothenburg, 41345 Gothenburg, Sweden; tanmoy.mondal@gu.se; 4 Department of Biotechnology, College of Science, Taif University, P.O. Box 11099, Taif 21944, Saudi Arabia; maorabi@tu.edu.sa

**Keywords:** SARS-CoV-2, COVID-19, variant, molecular dynamics, double mutant, delta variant, kappa variant

## Abstract

The recent emergence of novel SARS-CoV-2 variants has threatened the efforts to contain the COVID-19 pandemic. The emergence of these “variants of concern” has increased immune escape and has supplanted the ancestral strains. The novel variants harbored by the B.1.617 lineage (kappa and delta) carry mutations within the receptor-binding domain of spike (S) protein (L452R + E484Q and L452R + T478K), the region binding to the host receptor. The double mutations carried by these novel variants are primarily responsible for an upsurge number of COVID-19 cases in India. In this study, we thoroughly investigated the impact of these double mutations on the binding capability to the human host receptor. We performed several structural analyses and found that the studied double mutations increase the binding affinity of the spike protein to the human host receptor (ACE2). Furthermore, our study showed that these double mutants might be a dominant contributor enhancing the receptor-binding affinity of SARS-CoV-2 and consequently making it more stable. We also investigated the impact of these mutations on the binding affinity of two monoclonal antibodies (Abs) (2-15 and LY-CoV555) and found that the presence of the double mutations also hinders its binding with the studied Abs. The principal component analysis, free energy landscape, intermolecular interaction, and other investigations provided a deeper structural insight to better understand the molecular mechanism responsible for increased viral transmissibility of these variants.

## 1. Introduction

Today, the entire world is struggling with coronavirus disease 2019 (COVID-19), a pandemic caused by severe acute respiratory syndrome coronavirus 2 (SARS-CoV-2) [1]. This virus has rapidly spread worldwide, and subsequently, its infectivity has been reported from every part of the world [2,3,4]. Accounting for several millions of worldwide deaths, this viral disease has presented a significant challenge. One of the main attributes of viruses is their ability to mutate frequently [5,6]. Therefore, the occurrence of new mutations affects the virulence and transmission of the virus [7,8,9].

**Figure 1:**
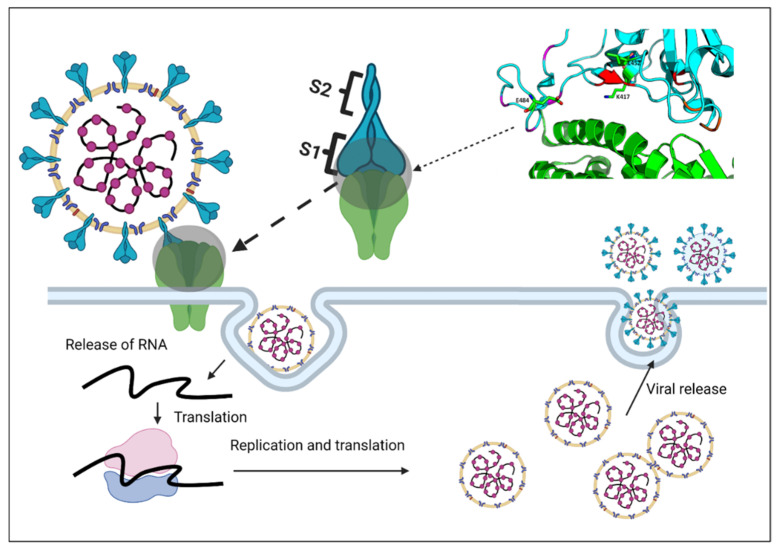
The viral entry mechanism of SARS-CoV-2 via the interaction of its spike protein with the host ACE2.

The Spike (S) protein is an essential part of SARS-CoV-2 as it mediates interaction with the human cells and is the target for most vaccine and therapeutic antibodies (Abs) [10,11]. Similar to other coronaviruses, the S protein of SARS-CoV-2 is responsible for its binding and attachment to the host cell-surface receptor. This surface glycoprotein consists of two functional domains: S1 and S2 [11,12]. S1 is located on the cell surface; its receptor-binding domain (RBD) mainly interacts with the host cell receptor while the S2 domain is located inside the membrane of coronavirus, mediating membrane fusion (Figure 1) [13,14,15]. The viral entry of SARS-CoV-2 within the host cell is mediated by binding its surface S protein to the host angiotensin-converting enzyme 2 (ACE2) [16]. The S protein's RBD is the region responsible for the attachment [17]. The viral entry and its propagation in the human host are well depicted in Figure 1.

Several studies have confirmed the role of this RBD–ACE2 interaction in the viral entry process [18,19,20]. In addition, the impact of naturally selected mutations within RBD on viral entry, infectivity, pathogenesis, and immune escape has been well documented [7,21,22].

Mutation in S1 or S2 region may result in variations in virus infectivity into the host cell [23,24,25]. The recently reported variants of SARS-CoV-2 possess higher transmissibility [22]. These recent emergences of new SARS-CoV-2 variants have increased the viral transmission and threatened the effectiveness of vaccines and other small molecule inhibitors designed explicitly for the COVID-19 pandemic. These newly reported “variants of concern” harbor mutations that confer increased viral transmissibility or immune escape [8,26]. The mutation-induced conformational changes in these new reported variants might also make the vaccines or neutralizing antibodies (nAbs) ineffective [21,22,26,27]. The recently reported SARS-CoV-2 variant B.1.617 (delta and kappa) are responsible for the steep rise in the number of COVID-19 cases and deaths in India [28,29,30]. The emergence of these new SARS-CoV-2 variants is believed to be highly responsible for several million new infections, leading to thousands of new deaths within a few weeks [29].

The SARS-CoV-2 B.1.617 lineage, first identified in India, has become dominant in several parts of the globe [31,32,33]. This lineage has been classified into three sublineages viz. B.1.617.1, B.1.617.2, and B.1.617.3 [34]. All the sublineages are found to be harboring diverse mutations within the RBD of the S protein [32]. It is believed that the rapid spread of the B.1.617 variant in India might be due to the presence of some essential point mutations within the RBD, which might be promoting the cellular entry of the virus, thereby infecting a broader range of target cells [35]. These mutations are also reported to be mainly responsible for their increased immune evasion potential [36]. The SARS-CoV-2 S protein of the B.1.617.1 (kappa) variant harbors a total of eight mutations [37]. Of these eight mutations, seven are located within the S1 region, while one is located within the S2 subunit. This variant harbors two mutations within the RBD (L452R, E484Q), the region responsible for the viral entry [37]. This double mutation is also carried by the B.1.617.3 as well [32]. Here in this study, we have investigated the impact of the double mutation (L452R + E484Q and L452R + T478K) carried by the B.1.617.1 (kappa variant) and the B.1.617.2 (delta variant), respectively, on the binding propensity of SARS-CoV-2 S protein with the human ACE2. Both double mutants (dm) investigated in this study are reported to be originating from India and are primarily responsible for the surge in COVID-19 cases in India [38]. Delta variant rapidly spread and became dominant in India and other countries [32,39,40]. The impact of delta variant, classified as variants of concern (VOC) by the World Health Organization (WHO), has been well reported [41,42,43]. The findings of this study demonstrate the dominant impact of double mutations carried by the kappa and delta variants on the binding capabilities between S and ACE2. The double mutations carried by these variants affected the microenvironment of the S–ACE2 interface region, thereby promoting the stability of the complex. The presence of the studied double mutations was also observed to be restricting the Ab binding. This further challenge the possibilities of effective vaccine design and therapeutics. The detailed investigation of the impact of delta and kappa variants envisage their role in promoting the virus’s cellular entry, thereby making it an essential factor for a higher infectivity rate.

## 2. Materials and Methods

### 2.1. Model Building and Optimization

The crystal structure of the SARS-CoV-2 S receptor-binding domain bound with ACE2 was fetched from the RCSB (PDB ID 6M0J) [44]. The structure was modeled for the missing sidechain atoms and minimized. For the kappa variant, the L452 and E484 residues of the SARS-CoV-2 spike receptor-binding domain (wild) were mutated to 452R and 484Q, respectively. The L452R and T478R substitutions were performed for the delta variant.

The structure and binding mode for the neutralizing Abs 2-15 and LY-CoV555 were retrieved from the RCSB (PDB Id: 7L5B and 7KMG) [45,46]. Mutant spike complexes in conjugation with the Abs were generated and optimized.

### 2.2. Molecular Dynamics Simulation

All the complexes (wild and the two variants) were subjected to MD simulations. The MD calculations were performed on GROMACS 2020.4 package using the CHARMM27 force field [47]. Initially, both complexes were solvated within the TIP3P cubic solvation box with a 10 Å periodic boundary. To satisfy the electro-neutrality of the systems, Na+ and Cl− ions were added. Both systems were subjected to energy minimization using the steepest descent integrator. The energy minimized model was subjected to NVT and NPT ensemble for 100 ps to stabilize the system at 300K temperature and 1 bar pressure. The equilibrated system was further subjected to extended molecular dynamics simulation for 200 ns.

The structures for nAbs in a complex with complete S protein were minimized and simulated for 100 ns each. We followed the same molecular dynamics protocol and parameters as mentioned above for the RBD-ACE2 complex. Various parameters such as hydrogen bond, the distance between the atoms, contact surface area were calculated using the inbuilt GROMACS modules. The average trajectories were extracted, and the center of masses was calculated for RBD and ACE2 subunits to obtain the lateral and angular shifts (tilt angle).

### 2.3. Principal Component Analysis

Principal component analysis (PCA) was performed and analyzed to investigate the collective motions of the wild type (wt) and dm. The covariance matrix C was calculated using the following Equation (1):

(1) $$C_{ij}=<\left(x_{i}-<x_{i}>\right)(x_{j}-<x_{j}>)>$$

where *x_i_* and *x_j_* are the instant coordinates of the *_i_*^th^ and *_j_*^th^ atoms of the systems, while <*x_i_*> and <*x_j_*> represents an ensemble average. The trajectories were analyzed for the relative motions about their center of masses.

### 2.4. Free Energy Landscape

The Free energy landscape (FEL) was analyzed to understand the stability and folding of wt and dm. The FEL was depicted as Equation (2):

(2) $$∆G\left(X\right)=K_{B}T\ln P\left(X\right)$$

where Boltzmann constant was denoted by *K_B_*, *T* is absolute temperature, while the probability distribution of the molecular system along the PCs is denoted by *P(X)*.

### 2.5. Binding Free Energy Analysis

We determined the binding free on the binary complexes using GMXPBSA 2.1, a Bash/Perl-based tool for MM/PBSA calculations utilizing structural ensembles generated of GROMACS trajectories. It calculates the binding free energies of the complexes [48].

This approach calculates the binding free energy (Δ*G_binding_*) according to the following Equations (3)–(5):

(3) $$∆G_{binding}=∆G_{MM\;\left(Potential\;energy\;in\;vaccum\right)}+∆G_{sol\;\left(solvation\;effects\right)}$$

where

(4) $$∆G_{MM}=∆G_{coulomb\;\left(electrostatic\;interaction\right)}+∆G_{Vdw}$$

and

(5) $$∆G_{sol}=∆G_{polar}+∆G_{nonpolar}$$

## 3. Results

### 3.1. Structural Analysis of the Wild and Double Mutant SARS-CoV-2 Spike Protein Complexes

The Crystal structure of the SARS-CoV-2 spike receptor-binding domain bound with ACE2 (PDB ID: 6M0J) was taken as the reference [44]. The reference complex structures along with the kappa (L452R, E484Q) and delta variant (L452R and T478K) models were subjected to two hundred nanoseconds of MD simulations. Various structural analyses were performed to study the structural impact of these mutations.

**Figure 2:**
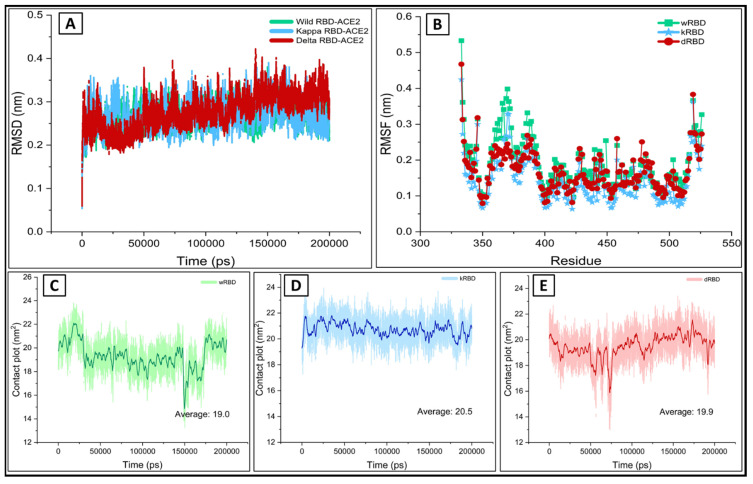
The molecular dynamics results of ACE2 in complex with the wild, kappa, and delta RBD during the 200 ns (**A**) The backbone RMSD of wtRBD (green), kRBD (blue), and dRBD (red) (**B**) The RMSF of wtRBD (green), kRBD (blue) and dRBD (red) in complex with the ACE2. The contact surface area of (**C**) wtRBD (green) (**D**) kRBD (blue), and (**E**) dRBD (red) structures of RBD bound to the ACE2.

The solvated system consists of 56,430, 56,440, and 56,665 atoms for the wt, kappa, and delta complex, respectively. The RMSD values relative to the initial crystal structures were determined, as shown in Figure 2A. The RMSD values for the wt receptor-binding domain (wtRBD) were found to be the lowest as compared with the values for selected variants, i.e., kappa(k)RBD and delta(d)RBD. A conformational shift was seen in the delta variant between 80–110 ns, which later stabilized. The average RMSD undulation amplitude was below 0.5 Å throughout most of the simulation time. The backbone RMSD of the ACE2 was stable in all the complexes with almost similar amplitude of fluctuations. The residual root mean square fluctuations (RMSF) values for the backbone atoms of the wt and mutant complexes were estimated (Figure 2B). A higher degree of fluctuation was observed in the native complex. However, the kappa variant witnessed the lowest level of fluctuations within the amino acid residues, suggesting the better stability of the kappa variant complex followed by the delta variant.

The interfacial binding contact surface area was estimated to be 19.0, 20.5, and 19.9 nm^2^ for wild, kappa, and delta, respectively (Figure 2C–E). The higher level of structural rigidity in both variants is attributed to the close and stable binding in the mutants. The studied parameters clearly indicate the increased binding interaction among the mutant complexes, which corroborates the previous reports on the increased virulence of these variants [49,50].

### 3.2. Essential Dynamics Outcome: Mutation Induced Local Structural Rigidity and Conformational Shift

**Figure 3:**
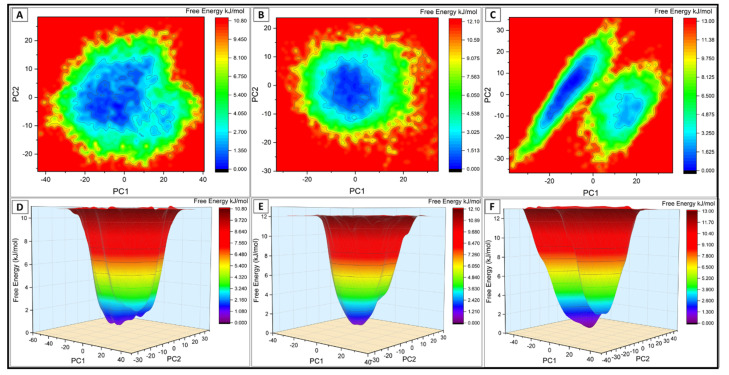
The 2D and 3D graphical representation of the free energy landscape of the (**A**,**D**) wtRBD–ACE2, (**B**,**E**) kRBD–ACE2, and (**C**,**F**) dRBD–ACE2 complexes.

PCA or essential dynamics is one of the dimensionality reduction techniques used to extract the principal motion in conformational ensemble generated by protein dynamics in collective coordinate space [51]. This statistical technique is significant for reducing the data complexity [52]. The PC1 and PC2, along with the corresponding free energy, were plotted (Figure 3). As shown in Figure 3, most of the simulation ensembles in the variants are concentrated to a narrow range of conformational space, indicating better stability and compact packing of the variant complexes.

**Figure 4:**
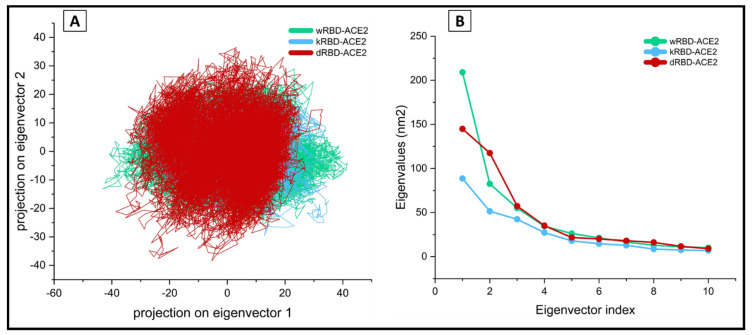
(**A**) The 2D eigenvector projection plot showing the differences in the dynamic fluctuations of the wtRBD (green), kRBD (blue), and dRBD (red) complexes of SARS-CoV-2 with the ACE2 (**B**) the plot for the eigenvalues for the top 10 selected vector.

The conformational sampling of tertiary structure for the wt, kappa, and delta in the essential subspace along eigenvectors 1 and 2 is shown in Figure 4A. The entire conformational space was classified into 2373 eigenvectors obtained from the diagonalization of the covariance matrix of the atomic fluctuations of each ensemble in the wt and the selected mutant protein simulations. The top ten vectors, along with their eigenvalues, were plotted. A fall in the eigenvalues was noted as expected. The first ten principal components accounted for more than 80% of the total motions observed during the simulation of RBD–ACE2 complex systems (Figure 4B). The eigenvector1 and eigenvector2 projections for both variants show a compact cluster of stable states, while a slight state deviation was noticed in the wt.

**Figure 5:**
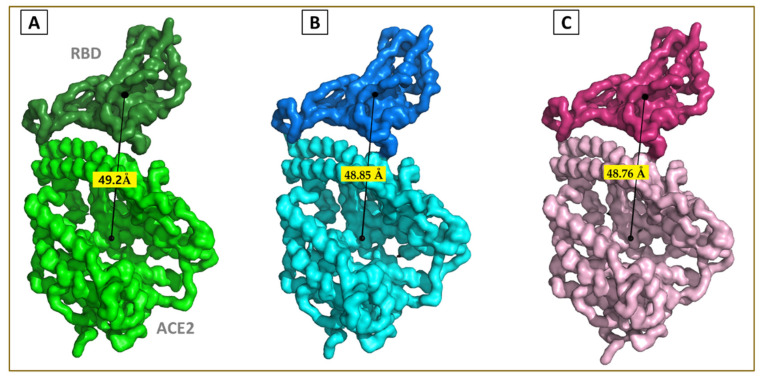
The average lateral drift between the COM of (**A**) wtRBD and ACE2, (**B**) kRBD and ACE2, (**C**) dRBD and ACE2.

Further, we analyzed the lateral drift and angular tilt across the domains. The average trajectories were extracted, and the center of masses was calculated for RBD and ACE2 subunits. The distance between the two centers of mass (COM) was found to be decreased from 49.2 Å (in the wt) to 48.85 Å (kappa) and 48.76 Å (delta) (Figure 5). The decrease in the magnitude of the distance between the COM for the variants suggests a more compact topology with more intermolecular interactions.

**Figure 6:**
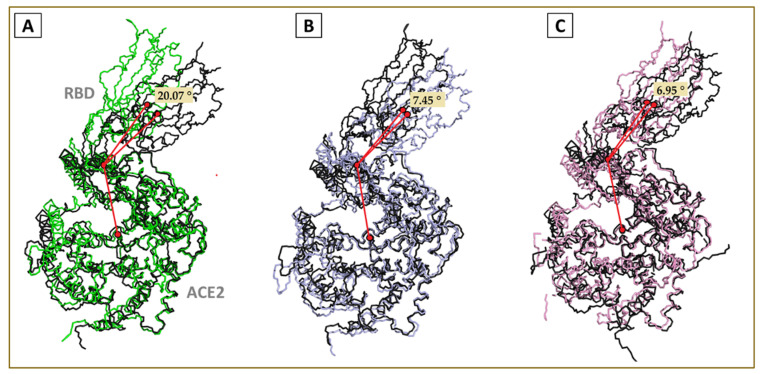
Characteristic relative angular twist of the (**A**) wtRBD–ACE2, (**B**) kRBD–ACE2, and (**C**) dRBD–ACE2 complexes.

We also analyzed the distinct trajectories in the largest eigenvector for angular tilt. Figure 6 shows the angular fluctuation between the COM for RBD, ACE2, and the least fluctuating hinge helix (HH) between residue 21 to 51 on the ACE2 protein. The wt complex was found to have the highest rotational freedom (20.07°) compared with that of kappa (7.45°) and delta (6.95°) variant complexes. This observation also favors the previous findings showing the impact of mutations increasing the binding of RBD toward the human ACE2.

### 3.3. Intermolecular Binding Free Energy Estimation

The intermolecular binding free energy change (ΔΔG) between the RBD (wt, kappa, and delta variants) in complex with the ACE2 was also calculated. The total free energy of binding, Van der Walls energy, and electrostatic energy of binding are illustrated in Table 1. The binding free energy change (ΔΔG) for the wtRBD–ACE2 complex was −51.96 Kcal/mol. The magnitude increased to −67.19 Kcal/mol and −64.58 Kcal/mol for the kappa and delta-bound ACE2 complexes, respectively. The higher negative magnitude in the ΔΔG shows the mutations carried by these variants significantly increased their binding affinity towards human ACE2. The occurrence of these point mutations stabilized the binding of delta and kappa variants towards ACE2 (Table 1). The Van-der Waals energy contribution is relatively constant; however, the variants’ electrostatic energy is highly increased.

### 3.4. Inter and Intra Molecular Interaction Analysis

**Figure 7:**
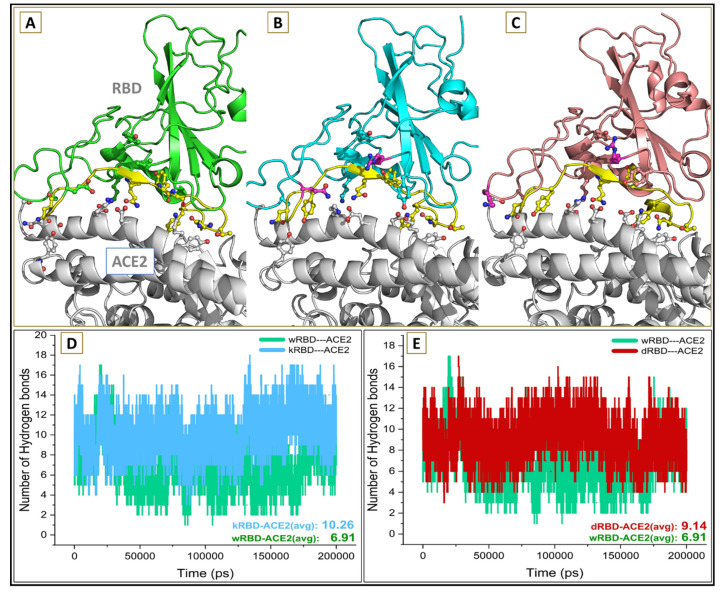
The interfacial binding region between (**A**) wtRBD–ACE2 in green, (**B**) kRBD–ACE2 in cyan and (**C**) dRBD–ACE2 complex in pink color. The average number of intermolecular hydrogen bonds between (**D**) wtRBD–ACE2 (green) and kRBD–ACE2 (cyan) and (**E**) wtRBD–ACE2 (green) and dRBD–ACE2 (red).

The total intermolecular hydrogen bonds between binary protein complexes during the course of the simulation were analyzed to investigate the extent and nature of interactions between RBD and ACE2 protein in wt and selected variants. The average number of hydrogen bonds at the interfacial binding surface of the kappa and delta variant were 10.26 and 9.14, respectively (Figure 7), which is relatively higher compared with the wt (6.91).

The role of critical residues involved in binding SARS-CoV-2 S protein to the human ACE2 was explored. Residues 417Lys, 487Asn, 493Gln, 495Tyr, and 505Tyr on the RBD surface were involved in hydrogen bonding interactions in the wt complex (Table 1). Apart from these residues, the kappa variant has seven additional residues (449Tyr, 476Gly, 496Gly, 498Gln, 500Thr, 501Asn, 502Gly) and the delta variant has two additional residues (500Thr, 501Asn), found involved in forming the intermolecular hydrogen-bonded interactions.

**Figure 8:**
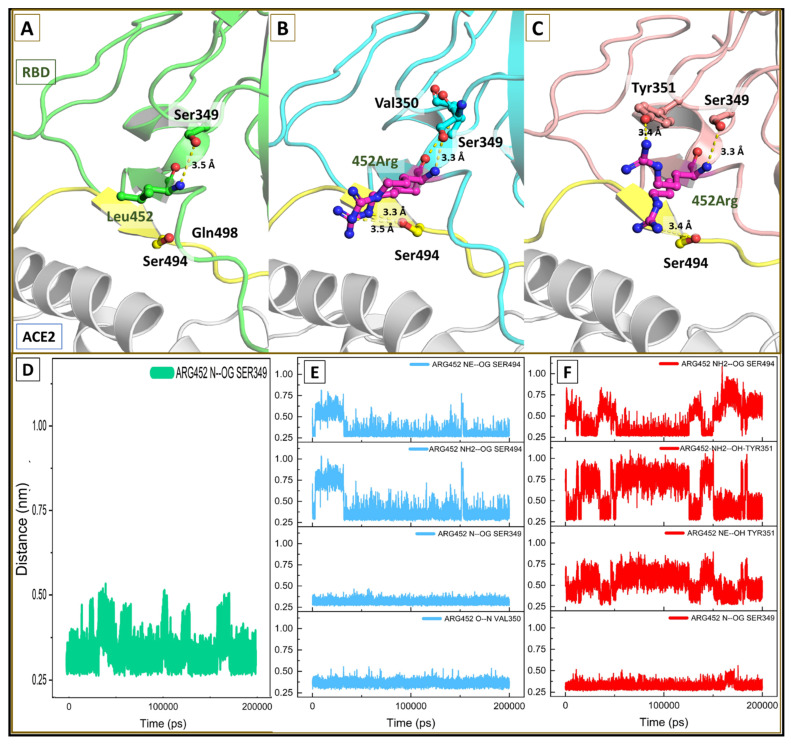
Binding conformation of mutant residue 452. (**A**) Leucine 452 making backbone hydrogen bond interaction with Ser349; (**B**,**C**) showing hydrogen-bonded interaction of 452Arg in kRBD and dRBD; (**D**–**F**) plot for hydrogen bond distance through the course of simulation in wtRBD, kRBD and dRBD.

The mutant residue was found to have a cascading effect on the interfacial surface of the RBD. The backbone nitrogen is making a consistent hydrogen-bonded interaction with the hydroxyl group of the Ser349 in the wt and both variants as well (Figure 8A–C). The polar side chain in the kappa and delta variant forms some additional hydrogen-bonded interactions with the hydroxyl oxygen on Ser494 (Arg452 NH2/NE-OG Ser494). The bond distance through most of the dynamics time was identified to be <=3.5A. In the delta variant, the basic side chain of mutant residue Arg452 orients itself in two different configurations and is stabilized by hydroxyl group of Tyr351 and Ser494 (Arg452 NH-OG Ser494 and Arg452 NH-OH Tyr351).

**Figure 9:**
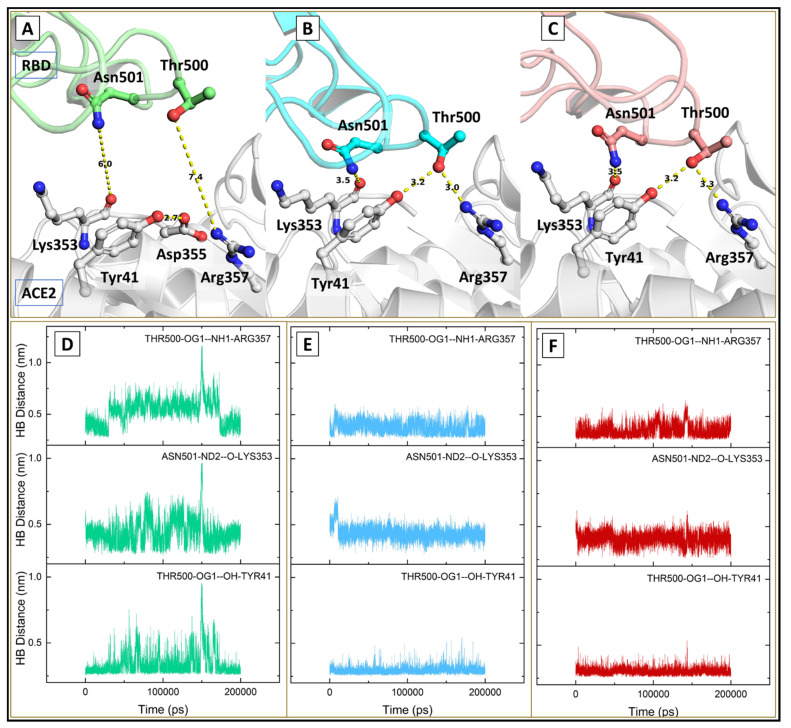
Intermolecular hydrogen bonds at the binding interface (**A**–**C**) showing hydrogen-bonded interactions in the wild (green), kappa (cyan), and delta (red) respectively; (**D**–**F**) plot for hydrogen bond distances during simulation time.

**Figure 10:**
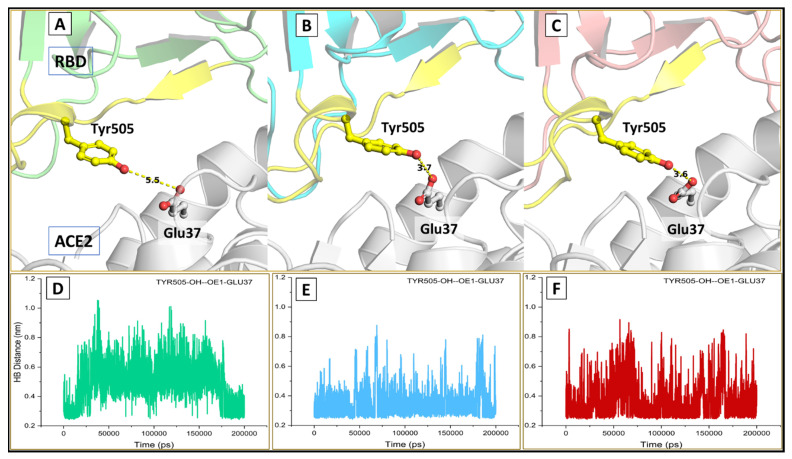
Intermolecular hydrogen bonds at the binding interface (**A**–**C**) showing hydrogen-bonded interactions at position 505 in the wild (green), kappa (cyan) and delta (red) respectively; (**D**–**F**) plot for hydrogen bond distance.

We also analyzed the comparative interactions at the complex interface and found that in both variants, three additional interactions (Thr500-OG1-NH1-Arg357, Asn501-ND1-O-LYS353, and Thr500-OG1-OH-Tyr41) were well maintained throughout the simulation period (Figure 9). Another consistent hydrogen bond interaction between Tyr505-OH-OE1-Glu37 was present discriminately in the kappa and delta variant; however, it was missing in the wt complex. These interactions add to the binding potency of the variants and may be attributed to further strengthening the binding ability in the mutant complexes (Figure 10). The higher number of hydrogen bonding residues reflects better stability and compactness of the mutant complexes.

### 3.5. Interaction between Neutralizing Antibody and RBD

**Figure 11:**
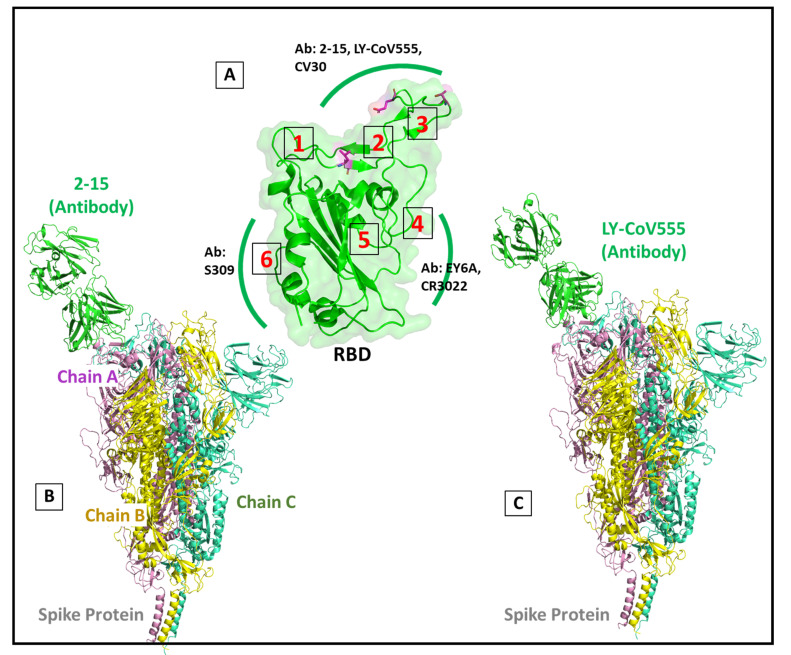
Diagrammatic representation depicting different antibody binding regions on the RBD. (**A**) ribbon and surface representation of RBD showing different antibodies targeting different regions. The mutant residues are colored in magenta (**B**,**C**) ribbon representation showing antibody 2-15 and LY-CoV555 orientated to RBD.

Several Abs are reported to be interacting with the RBD, and their positional clustering analysis data suggest five distinct regions of attachment on the RBD (Figure 11A). To find the effect of selected mutations on the binding response of nAbs, we performed molecular dynamic studies on the wild-type RBD–Antibody (wtRBD–Ab), delta RBD–Antibody (dRBD–Ab), and kappa RBD–Antibody (kRBD–Ab). Here we investigated two different monoclonal Abs that specifically target regions 2 and 3 lying close to the mutation site on the RBD of the S protein (Figure 11A).

**Figure 12:**
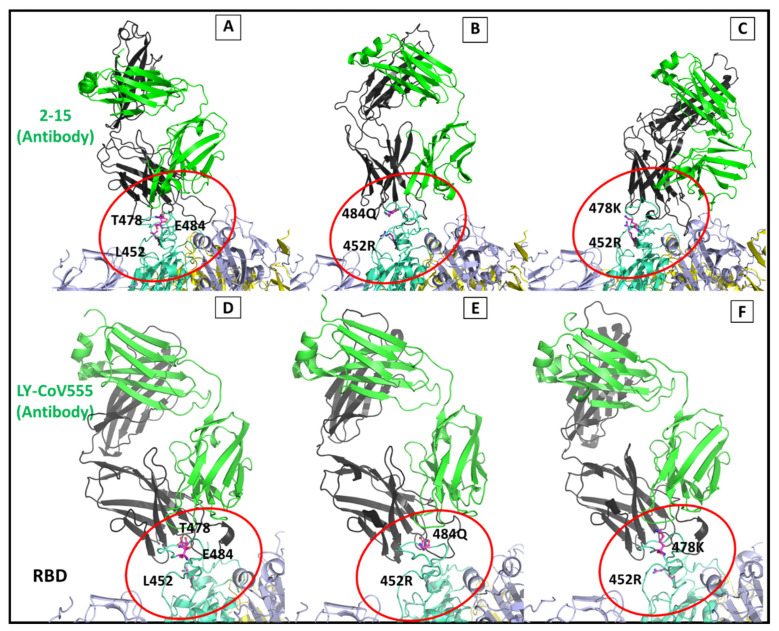
Diagrammatic representation of spike protein conjugated with antibody. (**A**–**C**) MD structure showing spike-2-15 antibody interface in wild, kappa and delta variants; (**D**–**F**) MD structure showing spike-LY-CoV555 antibody interface in wild, kappa and delta variant. The red circle highlights the interfacial site and the mutant residues.

To account for the changes in the binding pattern in the variants, we build the complete complex system around 6 Å distance on the Abs, which included the other two subunits of the homotrimer S protein, as shown in Figure 11. The S protein consists of three chains (A, B, and C). The neutralizing Abs orient themselves at the RBD domain (Figure 11B,C), with mutant residues depicted inside the red circle (Figure 12A–F). SARS-CoV-2 neutralizing Ab 2-15 and LY-CoV555 (bamlanivimab) were selected for the mutational analysis. The complex system was exceedingly large, with 80,367, 80,325, and 80,349 atoms in wt, kappa, and delta mutant complexes, respectively. Our study found that the mutations in the delta and kappa variants of SARS-CoV-2 decrease the binding affinity of the selected Abs. We also observed the changes in the loop conformations at the interface in the dRBD–2-15 complex. The Ab binding region is shifted with the lighter chain of Ab, making hydrophobic interactions with the loop residues on the chain B subunit of the S protein.

We determined the binding free energy for RBD–2-15 and RBD–LY-CoV555 complexes (Table 2). The energy values for the wt, kappa and delta complexed with Ab 2-15 were found to be −50.82, −44.76, and −41.64, and for Ab LY-CoV555, the values were −68.44, −30.84, and −57.08, respectively. The electrostatic energy contribution to the binding affinity calculated by the MM force field is −19.24, 337.05, and 146.18 for LY-CoV555 and 255.29, 689.15, and 731.41 for 2-15 nAbs.

The light chain region of the Abs was found to interact with the homologous B-chain of the S trimer protein. In both variants, replacing the hydrophobic residue at the 452 positions (Gln) with a polar residue (Arg) sterically restricted the binding of the studied Abs. We found that LY-CoV555 showed a strong binding affinity towards the wtRBD; however, the affinity was reduced in the selected variants. The binding free energy change (ΔΔG) was calculated. It was found that the ΔΔG of the wtRBD–LY-CoV555 complex was −68.4 Kcal/mol, which was reduced to −30.8 and −57 Kcal/mol in the kappa and delta variants, respectively. These findings show the importance of L452, T478, and E484 in stabilizing the interaction of LY-CoV555 with the RBD. The occurrence of L452R and E484Q mutations in the kappa variant severely impacted the affinity of LY-CoV555. In the kappa variant, the binding of LY-CoV555 is escaped. The delta variant, which carried L452R and T478K mutations, also demonstrated a reduced affinity towards the LY-CoV555.

## 4. Discussion

COVID-19, the pandemic disease which has shaken the global medical settings, has impacted a significant proportion of the worldwide population. Despite being the main research focus accounting for massive research investment, searching for an effective agent to combat this infection remains challenging [53,54]. SARS-CoV-2, the etiological agent of this pandemic, binds to host ACE2, facilitating the viral entry [55,56]. This interaction of SARS-CoV-2 S-ACE2 is the first checkpoint. The emergence of novel SARS-CoV-2 variants has further added the hurdle toward effective therapeutic development [57,58]. B.1.617 [30,59], a recently reported SARS-CoV-2 variant, seemingly responsible for a steep increase in the global pandemic cases, has been studied.

Several studies have reported mutations within the S protein and their impact on virulence [31,60,61,62]. The dual mutations (L452R + E484Q and L452R + T478K) harbored by the novel variants (kappa and delta) were found to be responsible for the higher virulence [63]. Our study demonstrated that the presence of double mutations carried by the kappa and delta variants within the RBD on S protein of B.1.617 were responsible for the increase in binding capability with the human ACE2 and further established the structural basis behind it. The RBD lies between residues 331–524 of the S1 domain on S protein [64] consists of an antiparallel beta-sheet with five strands sandwiched between short helices and loops on either side. Most of the structure is in the flexible loop conformation. The interfacial binding surface (IBS) of the RBD that is involved in binding to the ACE2 receptor of the host involves mainly the loop conformation with residues Arg403, Glu406, Arg408, Gln409, Gly413, Gln414, Thr415, Gly416, Lys417, Ile418, Ala419, Asp420, Tyr421, Gly446, Tyr453, Leu455, Phe456, Ser459, Asn460, Leu461, Ala475, Gly476, Ser477, Phe486, Asn487, Tyr489, Gln493, Gly496, Gln498, Thr500, Asn501, Gly502, and Tyr505. Interface region 1 from Val483 to Tyr505 (IR1) is highly flexible and contains the mutant residue 484Gln. The other two mutant residues, 452Arg and 478Lys, are located on a loop adjacent to the IR1. The role of these residues at 484 and 452 in the binary complex formation are documented in previous studies [65,66]. This study also determined the effect of kappa and delta variants on Ab binding.

### 4.1. Molecular Dynamics Trajectory Analysis

The solvated systems for the wt and kappa variants were equivalent, while it was considerably higher in the delta variant. This occurred due to the introduction of a charged residue at the protein’s exposed surface, which attracted several solvent molecules. The RMSDs relative to the initial crystal structures were stable in all three systems. Compared with the wt, the RMSD values were higher for the selected variants as expected. Interestingly, the residual RMSF for the kappa and delta variant was lower in contrast to the wt. Further, the values for the interfacial site contact surface area were greater for the studied variants. These results distinctly exhibit the changes in conformational state of the secondary structures in the variants, which might have led it to attain a more stable orientation when complexed with the ACE2 with increased binding propensities of the kappa and the delta variants towards the ACE2 receptor.

### 4.2. Essential Dynamics Analysis of the Molecular Trajectories

Essential dynamics of the simulated trajectory identified a total of 2373 discrete vectors. Almost all (>80%) of the meaningful variances associated with the wt, kappa, and delta proteins were concentrated on the top ten principal components. The free energy contour plot (Figure 3) for PC1 and PC2 showed the least deviation among the ensembles for kappa and delta variants. The delta variant occupied a slightly different conformational space manifesting two less distant energy peaks of unique and distinct conformational class. Further analysis of the classified vectors for the phase space distribution properties of the motions described by the selected EVs revealed a narrow and concentrated conformational space in the mutant systems. A slight deviation is evident in the wt complex.

The analysis of the average ensemble from the largest vector in all the three complex systems revealed a lateral drift in the relative COMs, resulting in reducing the distance between the individual protein masses compared with the wt complex. The binary mutant complexes were more closely packed. Subsequently, we scrutinized the angular degree of freedom. The trajectory analysis revealed the characteristic twisting motion of the two proteins at the interface along the stable hinge helix (HH) on the ACE2 protein. The wtRBD–ACE2 binary complex had standard angular freedom of about 20.07° (Figure 6).

In contrast, the kappa and delta RBD–ACE2 complexes showed restricted angular freedom with the twist angle reduced to only 7.45°. The delta complex showed an angular twist of 16.45° within the initial 10 ns of the simulation phase, which stabilized and remained consistent at 6.95° throughout the rest of the simulation time, making the binary complex in a more compact and stable conformation. This behavior may have surfaced due to the angular rearrangement between the RBD and ACE2 to accommodate the bulky side chains of 452Arg, 484Gln, and 478Lys residue in the mutant proteins. Based on these observations at this stage, we anticipated a considerably increased number of interactions at the binding interface in the variants.

### 4.3. Intermolecular Affinity Analysis

To gain further insight into the structural aspect of this outcome, the detailed interactions at the binding site were studied. Initially, we examined the binding interaction of the mutant residues. Apart from the consistent hydrogen bond interaction between Ser349 and residue at 452 in the wt and mutants, some additional polar interactions were witnessed in the variants. The polar side chain at 452 in kappa and delta variant forms additional hydrogen bond interactions with the hydroxyl oxygen on Ser494. In the delta variant, the basic side chain of mutant residue Arg452 orients itself in two different configurations and is stabilized by hydroxyl groups of Tyr351 and Ser494. These additional interactions formed by the mutant residues stabilized the interfacial binding loop in the variants. The effect cascaded down, and interestingly, we found that the kappa and delta variants demonstrated lower RMSD, lower twist angle, and a higher contact surface area in the mutant complexes. Due to the restricted movement of the IR1, the orientation of the residues on it has taken a more favorable conformation to interact with ACE2 (Figure 3C–E). The hydrogen bond interaction 505Tyr OH—OE 37Glu (2.6 Å) was absent in the wt complex; however, it was found to be consistently present in the mutant complexes throughout most of the dynamic period. Likewise, 498Gln OE1—NZ 353Lys (3.4 Å) and 452Arg NH2—494Ser OG (3.8 Å) in the kappa, and 452Arg NH2—OH Tyr351 (3.4 Å), 502Gly N—O 353Lys (3.0 Å) and 500T OG—OD 355D (3.1 Å) were found to be making a hydrogen bond with the residues on ACE2 in most of the dynamic states compared to the wt complex. These additional hydrogen bonds between ACE2 and RBD in dm imparted an enhanced binding affinity responsible for better stability and anchoring of dm RBD within the ACE2 groove. Furthermore, the binding free energy values between the wtRBD–ACE2 complex were estimated to be less favorable than the kappa and delta variant complexes. The higher interaction free energies in the mutant complexes are attributed to the increased number of additional consistent hydrogen-bonded interactions that were not found throughout most states in the molecular dynamic simulations in the wt.

All the above in-depth comparative structural analysis of the binary complexes indicates the close, stable, and enhanced interfacial interactions as a dominant impact of the kappa and the delta variant. The study found that the selected variants may be a dominant contributor enhancing the receptor binding of SARS-CoV-2, hence making it more virulent.

### 4.4. Neutralizing Antibody Complex Analysis

The nAbs recognize RBD, or other regions on the S glycoprotein, directly or indirectly interfering with the ACE2 interaction [67]. Previous studies have shown that different Abs target different regions on the S protein [68,69]. The epitome mapping studies on the Abs specific to S protein showed that 21% of the 377 epitopes were from RBD [70]. Further clustering analysis on 80 monoclonal Abs has identified five distinct RBD regions where Abs select to bind [70]. We specifically selected SARS-CoV-2 nAbs 2-15 and LY-CoV555 for the mutational analysis. The reason for choosing these two Abs was their selective binding on the region close to the mutant residue on RBD of the S protein. The S protein is a homo-trimer protein with each subunit interacting with the ACE2 receptor. The second and the third chain lies within 5 Å distance to the complexed Ab. Therefore, we selected the entire trimer of the S protein to avoid any unanticipated bias arising due to an incomplete system. The system size formed was extremely large but requisite. The interfacial binding region between RBD and nAb on the S protein majorly consists of hypervariable loop conformation. As shown in Figure 11, the mutations are located in the binding region to the Abs. The mutations in kappa and delta variants at the interfacial binding surface with the bulky side chain and altered polarity presented different spatial and conformational surfaces to the interacting Abs. Due to these structural changes in the interfacial binding surface residues, the light chain region of the 2-15 Ab was found to interact extensively to a region distant from the actual binding surface, as seen in the wt crystal complex.

The binding free energy values for the RBD–Ab complexes in wt, kappa and delta variants were compared. The value for the energies clearly shows the decreased affinity of the Abs towards the variants. The 2-15 Ab, which targets the common interfacial surface on the RBD domain as with human ACE2 protein, has the least affinity for the delta variant. Compared with the wtRBD–Ab complex, the electrostatic energy contribution to binding free energy for the kRBD–Ab and dRBD–Ab complexes was highly reduced. Additional interaction between residues on the light chain and mutant residues on the S protein was found in the delta variant. Increased hydrophobic interaction with offsite residues (other than the native binding site) is seen in the delta variant, which is attributed to the extended off-target binding of the light chain of the Ab to the adjacent homologous subunit of the S trimer protein. Overall, the binding free energy of binding in the kappa and delta mutant strains is reduced compared with that of wtRBD-neutralizing Ab complexes. The study’s finding shows significantly reduced interactions of the selected Abs, clearly suggesting a possible immune escape mechanism by both the kappa and the delta variants.

## 5. Conclusions

This study concludes that the occurrence of kappa (L452R, E484Q) and delta (L452R + T478K) variants were more stable than the wt SARS-CoV-2 S protein. The variants were observed to be making a few additional hydrogen-bonded interactions which caused conformational changes at the binding interface. The relative angular freedom between the RBD and ACE2 protein is reduced in the kappa and delta RBD–ACE2 complexes. The larger contact surface area and higher intermolecular interactions enhance the affinity of mutants toward the ACE2. It was also observed that the new variants had reduced interactions with the nAbs compared with the wt. Our findings indicate that the local conformational shift at the interface region in mutant S protein results in a more stable, compact, with a higher contact surface area, a higher number of interactions, and lower negative interaction energies than the wt protein. These parameters might be responsible for the virus being more virulent. We observed the off-target binding of the Abs on the RBD of S protein with reduced Gibbs free energy in the kappa and delta variants, clearly suggesting a possible mechanism of immune escape by the kappa and delta variants.

## Figures and Tables

**Table 1:** The binding free energy components for the wtRBD, kRBD, and dRBD in complex with the ACE2.

Complex	DELTA G (Avg (Std. Err. of Mean) kcal/mol)	VDWAALS (Avg (Std. Err. of Mean) kcal/mol)	EEL (Avg (Std. Err. of Mean) kcal/mol)	DELTA G Gas (Avg (Std. Err. of Mean) kcal/mol)	DELTA G solv (Avg (Std. Err. of Mean) kcal/mol)
wtRBD–ACE2	−51.96 (0.52)	−83.02 (0.39)	−672.79 (2.42)	−755.82 (2.54)	703.85 (2.34)
kRBD–ACE2	−67.19 (0.57)	−79.75 (0.33)	−1081.61 (1.78)	−1161.36 (1.79)	1094.16 (1.81)
dRBD–ACE2	−64.58 (0.74)	−80.21 (0.39)	−1054.76 (3.18)	−1134.96 (3.08)	1070.38 (2.87)

**Table 2:** The interaction-free energies between the RBD (wtRBD, kRBD and dRBD) in complex with the ACE2 (in kcal/mol) for neutralizing antibodies 2-15 and LY-CoV555.

	DELTA G (Avg./Std. Err. of Mean)	VDWAALS (Avg./Std. Err. of Mean)	EEL (Avg./Std. Err. of Mean)	DELTA G Gas (Avg./Std. Err. of Mean)	DELTA G Solv (Avg./Std. Err. of Mean)
wtRBD–2-15	−50.82 (1.46)	−83.11 (2.22)	255.29 (4.42)	172.18 (5.18)	−222.99 (4.68)
kRBD–2-15	−44.76 (1.20)	−79.58 (1.23)	689.15 (5.54)	609.57 (5.77)	−654.33 (5.27)
dRBD–2-15	−41.64 (1.17)	−101.05 (1.37)	731.41 (6.35)	630.36 (6.11)	−671.99 (5.88)
wtRBD–LY-CoV555	−68.44 (0.93)	−79.42 (0.62	−19.24 (2.49)	−98.66 (2.56)	30.22 (2.34)
kRBD–LY-CoV555	−30.84 (0.89)	−75.48 (0.65)	337.05 (3.13)	261.57 (3.25)	−292.42 (2.91)
dRBD–LY-CoV555	−57.08 (0.75)	−78.26 (0.44)	146.18 (3.22)	67.92 (3.14)	−124.99 (2.99)

## Data Availability

Not applicable.
